# Resveratrol Suppresses Prostate Cancer Epithelial Cell Scatter/Invasion by Targeting Inhibition of Hepatocyte Growth Factor (HGF) Secretion by Prostate Stromal Cells and Upregulation of E-cadherin by Prostate Cancer Epithelial Cells

**DOI:** 10.3390/ijms21051760

**Published:** 2020-03-04

**Authors:** Tze-chen Hsieh, Joseph M Wu

**Affiliations:** Department of Biochemistry and Molecular Biology, New York Medical College, Room 147, Valhalla, NY 10595, USA; tze-chen_hsieh@nymc.edu

**Keywords:** resveratrol, prostate cancer, HGF, EMT

## Abstract

Cancer mortality is primarily attributed to metastasis and the resulting compromise of organs secondary to the initial tumor site. Metastasis is a multi-step process in which the tumor cells must first acquire a migratory phenotype and invade through the surrounding tissue for spread to distant organs in the body. The ability of malignant cells to migrate and breach surrounding tissue/matrix barriers is among the most daunting challenges to disease management for men in the United States diagnosed with prostate cancer (CaP), especially since, at diagnosis, a high proportion of patients already have occult or clinically-detectable metastasis. The interaction between hepatocyte growth factor (HGF) secreted by the stroma, with its receptor c-Met located in the epithelium, must occur for epithelial CaP cells to become migratory. We studied the effects of grape-derived phytochemical resveratrol on the transition of epithelial tumor cells from sedentary to a mobile, penetrant phenotype. A time lapse microscopy assay was used to monitor the acquisition of the migratory phenotype by resveratrol. The results show that resveratrol inhibits HGF-mediated interaction between the stroma and epithelium and suppresses epithelial CaP cell migration by attenuating the control of epithelial-to-mesenchymal transition (EMT).

## 1. Introduction

Metastatic prostate cancer (CaP) is a leading cause of death among men in the United States. An estimated 30% of first diagnosed CaP individuals show micrometastasis [1,2]. Moreover, in more than 50% of patients bone metastasis occurs eventually [3,4]. Metastasis and invasion are tumorigenic features that counter the efficacy of treatment for CaP; metastasis disseminates the tumor to ectopic sites and is a mitigating factor for therapies directed at the primary cancer site. Tumor invasion may result in significant comorbidity by compromising normal tissue and rendering tumor targeted approaches less feasible [5].

Several steps are involved in the acquisition of metastasis by primary epithelial tumor cells. These include epithelial-to-mesenchymal transition (EMT), an event that encompasses structural and functional reconfiguration of the local microenvironment through dynamic interplay between epithelial tumor cells and their adjuxtaposed neighboring cells. The EMT-programmed epithelial tumor cells can disseminate from the primary site to the bone marrow using two different conduits ― transport via the hematogenous (blood) vascular and/or lymphogenous (lymphatic) system. Interaction of disseminated epithelial tumor cells with endothelial cells in the bone marrow [6] is followed by resumption of proliferation in the distant, new sites as metastatic tumors. These changes as a whole suggest that metastasis is predicated on epithelial tumor cells acquiring motility by EMT that facilitates their exit from the primary site as the initiation of metastasis.

Acquisition of motility by prostate epithelial tumor cells via the induction of EMT is controlled by various cytokines and growth factors [7,8,9,10]. Among these, HGF, also known as scatter factor, regulates the acquisition of cell motility. HGF is synthesized and secreted by prostate stromal cells whereas it is absent in epithelial cells. HGF interacts specifically with its cognate receptor encoded by the proto-oncogene, c-Met [11,12,13], a transmembrane protein found on epithelial tumor cells harboring cytoplasmic tyrosine kinase activity to elicit signaling changes [14]. Negligible and high levels of c-Met expression occur in columnar epithelial and basal cells in benign prostate glands. However, in high-grade prostatic intraepithelial neoplasia (PIN) and carcinoma, more than 50% of foci stained uniformly for c-Met. The expression of c-Met in epithelial cells occurs in concordance with neoplastic progression [15,16,17,18]. Interaction of HGF with c-Met leads to increased kinase autophosphorylation, followed by the recruitment of signaling molecules such as Grb2, PI3-kinase, Stat-3, Gab1, etc., and subsequent cytoskeletal and Rho GTPase changes [19,20].

As mentioned, EMT is a requisite step for acquisition of motility by epithelial tumor cells. The induction of EMT involves transcriptional re-programming, notably the repression of epithelial genes (e.g., E-cadherin) and concomitant activation of mesenchymal genes (e.g., N-cadherin), alterations in the epithelial cell cytoskeleton structure, exemplified by the rearrangement of actin bundles, modulation of integrin, and expression and function of Rho family of GTPases. The Rho proteins regulate, respectively, the assembly of actin stress fibers and focal adhesions by Rho, induction of lamellipodia by Rac, and formation of filopodia by cdc42 [21]. Accordingly, for the migratory phenotype to be established in epithelial tumor cells, bidirectional signaling involving HGF secreted by the stroma impinging on c-Met in the epithelium is required. Silencing of HGF/c-Met using ribozymes reduces invasion and reverses malignancy [22,23].

We have been studying food-based approaches to prevent the progression of CaP to hormone refractory prostate cancer (HRPC), by focusing on the effects of grape-derived polyphenol, resveratrol. Using cultured CaP cells mimicking the androgen-dependent (AD) and androgen-independent (AI) states, we first reported that ≤ 25 µM resveratrol inhibited cell proliferation and down-regulated the expression of both intracellular and secreted prostate-specific antigen (PSA), a well-documented biomarker for CaP, in AD cells [24,25]. We further showed that resveratrol suppressed AI (DU145 and PC-3) cell growth [24]. These results suggest that resveratrol can block AD→AI transition, and the establishment of HRPC. A wealth of accumulated evidence points to a mutually-interactive and reciprocally-responsive relationship existing between epithelial tumor cells and adjacent stromal cells in the immediate microenvironment of the prostate, both in physiological settings and pathological states [10,13,26]. This new dynamic interplay between epithelial tumor cells and neighboring stromal cells is regarded mechanistically critical for the acquisition of EMT and epithelial cell growth, motility, migration, invasiveness, and metastasis [5,27,28,29]. Of note, the microenvironment niche playing an essential role for tumor growth and tumorigenesis is essentially a renaissance of the “seed and soil” hypothesis, first introduced by Paget in 1889 [30,31,32,33]. In this study, we examined the effects of resveratrol to elucidate the dynamic interplay between stromal and epithelial cells in the prostate microenvironment in the context of control of EMT and cell motility via the HGF/c-Met axis.

## 2. Results

### 2.1. Conditioned Media (CM) Prepared from Resveratrol-Treated Prostate Stromal Cells (PrSC) Inhibit Epithelial Prostate Cancer Cell Growth

**Figure 1:**
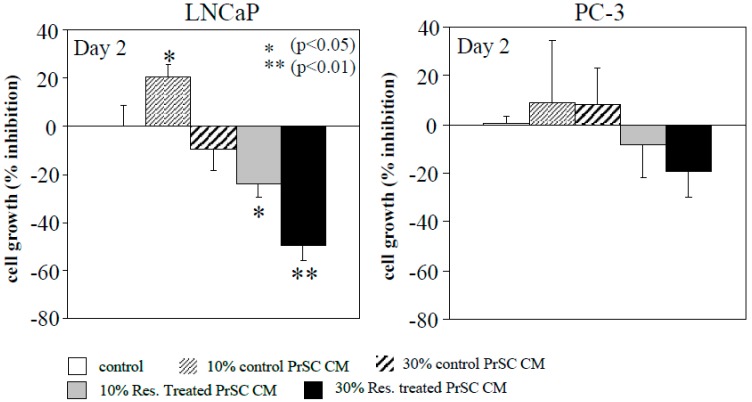
Conditioned media (CM) from prostate stromal cells (PrSC), with and without treatment by resveratrol, selectively inhibited growth of LNCaP cells. CM were obtained from control and 72 h resveratrol-treated PrSC, and added to LNCaP and PC-3 cells. CM-treated cells were harvested at 48 h and growth was monitored by trypan blue exclusion. Number of experiments: *n *= 6 for LNCaP cells; *n *= 3 for PC-3 cells.

CM elaborated by PrSC may appropriately recapitulate the spectrum of stimulatory and inhibitory diffusible factors, with cell-to-cell communication potential in regulating the growth of prostate epithelial tumor cells [34]. We tested whether the growth of epithelial CaP cells may be affected by paracrine-acting signaling molecules elaborated by PrSC. LNCaP and PC-3 cells maintained in 10% FBS supplemented RPMI 1640 media were separately treated with 10% and 30% CM obtained from day 2 untreated and 25 µM resveratrol-treated PrSC. At 48 h after addition of CM, cells were harvested and cell growth was monitored by trypan blue exclusion. Results show that growth of LNCaP cells was slightly stimulated and inhibited by 10% and 30% CM from untreated PrSC (Figure 1). By contrast, addition of 10% and 30% CM from 25 µM resveratrol-treated PrSC resulted in a significant, dose-dependent inhibition of LNCaP cells (Figure 1). When CM from untreated PrSC was added to PC-3 cells, no effect on cell growth was observed (Figure 1) while the addition of 10% and 30% CM from resveratrol-treated PrSC resulted in a small, insignificant decrease in PC-3 cell growth (Figure 1). As a control, we added CM from control and treated prostate epithelium cell (PrEC) which showed neither stimulatory nor inhibitory effect on growth of LNCaP or PC-3 cells (data not shown). These studies comparing CM from control and resveratrol-treated PrSC and showing that they exert differential growth modulatory effect on LNCaP and PC-3 cells support the thesis that resveratrol modulates signaling between stromal cells and stage-specific epithelial CaP cells (LNCaP, not PC-3) through paracrine-acting factors secreted into the culture media.

### 2.2. Tumor Cell Migration/Invasion is Significantly Inhibited by Resveratrol via Attenuation of Bi-Directional Signaling between PrSC and Epithelial Cells

**Figure 2:**
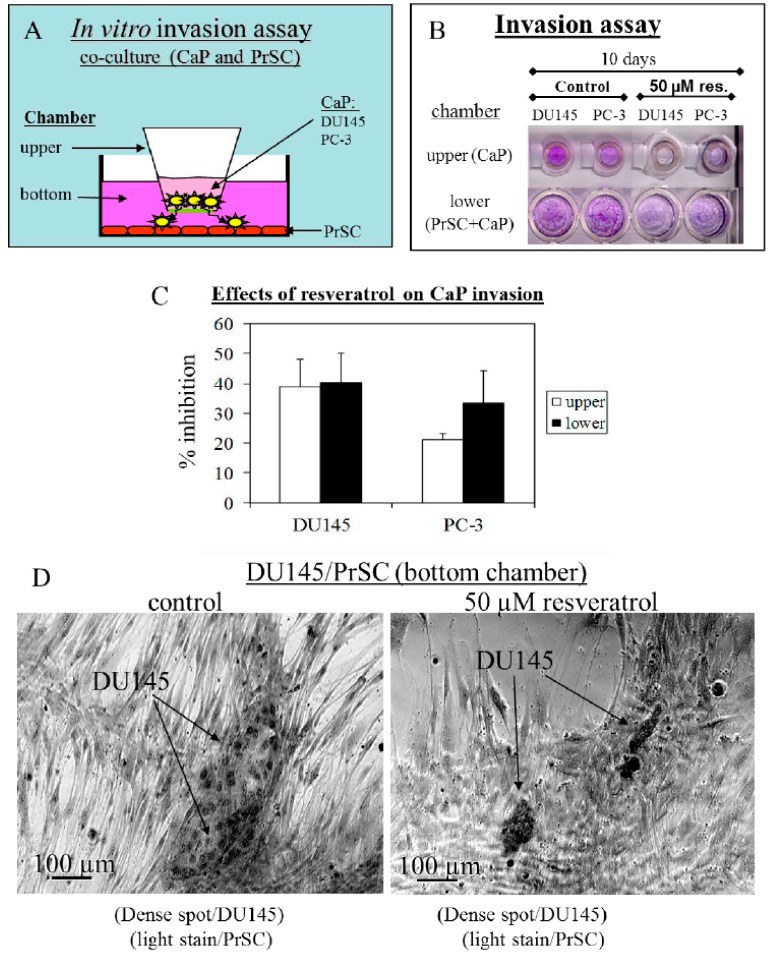
Effects of treatment by 50 µM resveratrol on interaction between prostate stromal and DU145 and PC-3 epithelial tumor cells. (**A**) Boyden chamber was used to study migration of epithelial tumor cells. (**B)** Photographs of crystal violet stained cells in the upper and lower chambers, respectively, support that resveratrol inhibits cell motility. (**C**) Quantitation of effects of resveratrol on CaP cell invasion was determined by extracting the dye-stained cells with 1% acetic acid and measuring OD_570_. (**D**) Intensity and morphology of dye-stained cells show resveratrol inhibits migration of DU145 cells across the Matrigel barrier.

To test that resveratrol affects PrSC impinging on migration/invasion of DU145 and PC-3 cells, we conducted studies using the Boyden chamber, with or without treatment by resveratrol, as schematically illustrated in Figure 2A. The results showed that migration/penetration of DU145 and PC-3 cells in upper chambers was inhibited by 39% and 21%, respectively, by resveratrol (Figure 2C). The results in the lower chamber, which represented both the migrated CaP and resident PrSC cells showed that penetration by DU145 and PC-3 cells was inhibited by 40% and 33%, respectively, compared to untreated wells (Figure 2C). This co-culture arrangement can also be used to monitor whether resveratrol affected the propensity of DU145 cells to penetrate the Matrigel barrier and become aligned with PrSC. Specifically, DU145 cells can be distinguished from PrSC based on stains retained by the two cell types. DU145 cells form large colonies and can; thus, be identified by the considerably darker stained appearance than the lightly-stained PrSC. Using this criterion, it can be seen in Figure 2D that treatment by resveratrol decreased the penetration of DU145 cells on the Matrigel, evident by the smaller and less intensely stained DU145 colonies. Taken together, these results suggest that resveratrol is capable of affecting stroma–epithelium interplay to inhibit migration/invasion in CaP cells, and disrupts the communication occurring between CaP and PrSC.

### 2.3. Control of CaP Cell Migration by PrSC Condition Medium (CM) and Assay of Secreted HGF in CM

**Figure 3:**
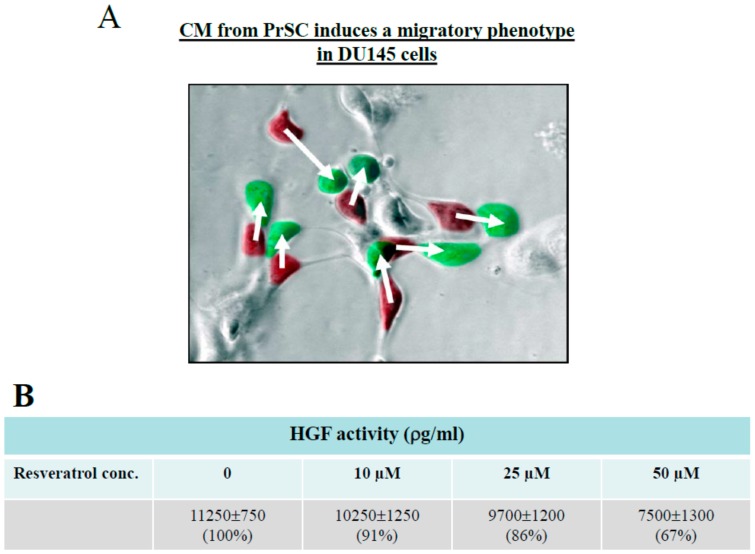
(**A**) Time lapse microscopy analysis of migration of prostate DU145 cells, in response to CM from PrSC. Two images, one taken at time 0 h and a second one take at time 2.4 h, were overlaid using Adobe Photoshop. Cells at 0 h were marked red, and cells at 2.4 h were marked green. The coordinates for each cell were obtained for each of the two time points, and the change in the distance for each cell was shown. (**B**) Effect of treatment by resveratrol for 24 h on secreted hepatocyte growth factor (HGF) by PrSC, assayed by ELISA.

DU145 cells have been used to study HGF-stimulate CaP cell scattering [35,36,37,38,39,40,41,42]. We found in our studies that DU145 cells grew in culture as small patchy clusters (without addition of CM from PrSC) or as scatter (with addition of CM from PrSC). We reasoned that the acquisition of motility by DU145 cells can be visually tracked as time-dependent morphological changes in growth, from patchy clusters to spread-out monolayers. Therefore, time lapse microscopy analysis was performed to capture the images at different time points following treatment by CM. Overlaid image between time 0 and 2.4 h showed that CM induced a migratory phenotype in DU145 cells (Figure 3A). Details of time lapse microscopy can be found in Supplementary Materials.

**Figure 4:**
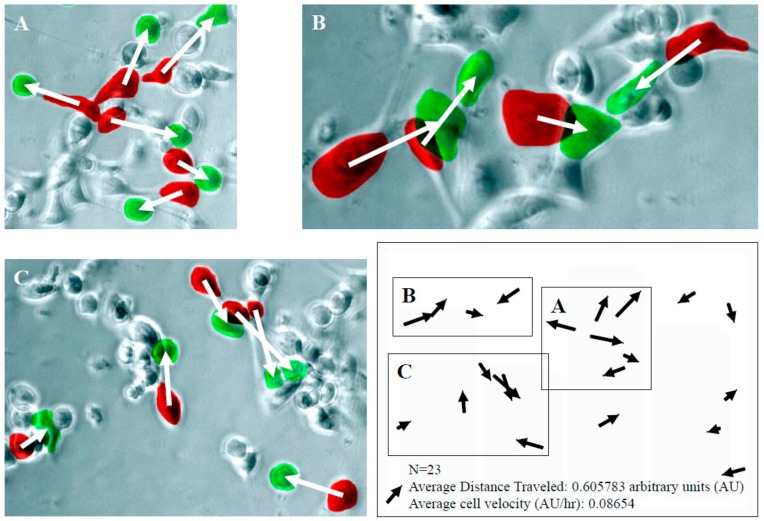
Detailed analysis of the rate of migration (in arbitrary units) of DU145 cells, in response to CM from PrSC. Two images, one taken at *t* = 0 h and a second one taken at *t* = 7 h were overlaid using Adobe Photoshop. Cells at *t* = 0 were labeled red, and cells at *t* = 7 were labeled green. 23 individual CM-treated DU145 cells located in three different microscopic fields, labeled as (**A**), (**B**), or (**C**) were used to calculate the average distance and rate of migration. The coordinates for each cell were obtained for each of the two time points and schematically shown in the lower right corner of Figure 4. The change in the distance migrated for each cell (*n* = 23) was calculated using the coordinates. The rate of cell migration was determined by the distance traveled as a function of time.

Results from Boyden Chamber studies (Figure 2) raise the possibility that suppression of migration by DU145 cells, in response to resveratrol, may be attributed to its ability to modulate growth factors elaborated by PrSC. To test this hypothesis, we focused on HGF, which has been reported to increase cell motility in a scatter assay and cellular invasive potential in a Matrigel invasion chamber assay using androgen-independent DU145 cells. Copious presence of HGF was found in cultured PrSC using ELISA (Figure 3B). Treatment with resveratrol for 24 h resulted in dose dependent suppression of HGF, with 50 µM resveratrol causing a ~33% reduction in secreted HGF. For comparison, HGF was undetectable in CM of PrEC (data not shown). Using time lapse microscopy analysis illustrated in Figure 3A, we monitored the CM-induced DU145 cell scatter/migration for 7 h and captured images at *t* = 0 h and *t* = 7 h, and calculated the average distance and rate of migration in DU145 cells treated with CM from 23 individual cells located in three different microscopic fields, labeled as A, B, or C. The coordinates for each cell were obtained for each of the two time points and schematically shown in the lower right corner of Figure 4. The change in the distance migrated for each cell (*n *= 23) was calculated using the coordinates. The rate of cell migration was determined by the distance traveled as a function of time. Results show that average cell distance traveled is ~0.6 arbitrary units (AU) and average rate of cell migration (AU/h) is ~0.087.

**Figure 5:**
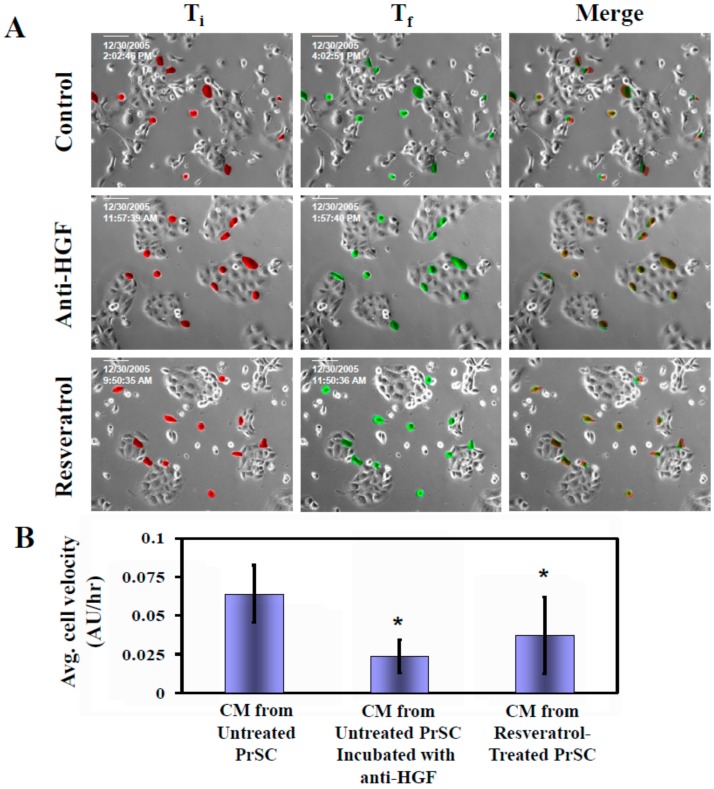
Effect of resveratrol and anti-HGF on CM-mediated migration of DU145 cells. (**A**) Time lapse microscopy analyses were performed to monitor the changes on DU145 cell migration for 2 h in cells treated with CM, with and without prior addition of excess of anti-HGF. Images were taken at initial time at time 0 (T_i_) and finish time at 2 h (T_f_). A Zeiss microscope equipped with Axiovert 2000 Imagining system (Carl Zeiss MicroImaging, Jena, Germany) was used to capture cell images at 20× magnification. Two images were merged as described in Supplementary Materials. (**B**) Calculated changes on the average cell velocity and average distance traveled in DU145 cells treated with CM, with and without prior addition of excess of anti-HGF (*p < 0.05). Asterisks (*) indicated statistically significant difference between treated groups compared with controls.

To test whether acquisition of migratory behavior in DU145 cells resulting from exposure to CM of PrSC is mediated by HGF, we added HGF-specific neutralizing antibody to CM derived from PrSC. Using the time lapse microscopy analysis approach illustrated in Figure 4, we monitored for 2 h and calculated the average cell velocity and average distance traveled in DU145 cells treated with CM, with and without prior addition of anti-HGF in excess. Results in Figure 5 show that average rate of DU145 cell migration was inhibited ~60% using HGF-neutralizing antibody. To investigate whether resveratrol significantly elicited decrease in HGF, the same cell velocity parameter was similarly determined in cells treated with CM prepared from resveratrol-treated PrSC. Figure 5 shows that average rate of cell migration was suppressed by ~40% using CM derived from resveratrol-treated PrSC, to a level comparable to suppression of secreted HGF in CM (Figure 3B). These results reinforce the notion that suppression of HGF secretion by resveratrol principally accounts for the attenuated migration observed in DU145 cells.

### 2.4. Effect of Resveratrol on Expression of E-Cadherin in DU145 Cells

**Figure 6:**
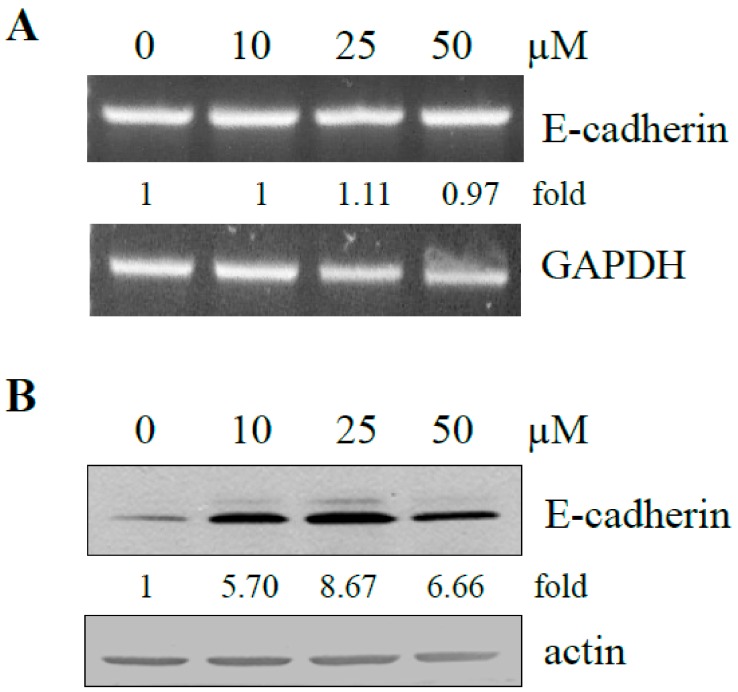
Effect of treatment by increasing dose of resveratrol on E-cadherin mRNA and protein expression. (**A**) DU145 was treated with 10, 25, and 50 µM resveratrol for two days. Changes in mRNA of E-cadherin were assayed by RT-PCR and quantified and expressed as a fold difference against GAPDH. (**B**) Changes on E-cadherin protein expression by resveratrol were analyzed by Western blot analysis. Intensity of specific bands from control and treated cells in panel B was each normalized to actin and expressed as fold of control.

EMT in vitro is initiated with the dissolution of tumor cells from colonies and the conversion of cancer cells to an independently motile phenotype. E-cadherin has been identified as a key player in the control of cell adhesion, motility, and invasive phenotype [43,44]. Our studies on cell scattering and cell migration strongly suggest that resveratrol is capable of altering EMT via inhibition of cell motility. To test if exposure to resveratrol affected E-cadherin expression in DU145, cells were treated with increasing dose of resveratrol. Control and treated cells were harvested and changes in E-cadherin mRNA and protein were measured by RT-PCR and western blot analysis. No change on E-cadherin mRNA was detected (Figure 6A), whereas significant up-regulation on E-cadherin protein expression (> 5-fold compared to untreated control) resulted from treatment by resveratrol (Figure 6B). These results suggest the resveratrol can reverse the invasive phenotype via upregulation of E-cadherin.

## 3. Discussion

The epithelium becomes significantly growth compromised when separated from the stroma, indicating that stromal–epithelial interactions play an important role in the control of prostatic epithelial cell growth [45,46,47,48,49]. This observation also implies that development of CaP might involve a loss of the regulated coordination in such interactions. Reversion of prostatic stroma to an “embryonic state” may induce inappropriate epithelial proliferation, with CaP as the ultimate clinical manifestation. As such, disruption or suppression of “embryonic state reversion” using the diet-based approach might serve to prevent the progression of CaP.

Anti-carcinogenesis effects of resveratrol first surfaced based on its demonstrated inhibition of the initiation and promotion of hydrocarbon-induced skin cancer in mice, as well as the progression of breast cancer in the same species [50]. For the past several years, we have been studying the mechanism of chemoprevention of resveratrol using CaP as a model and focusing on events in prostate epithelial tumor cells targeted by resveratrol. As noted, bi-directional interplay between the stroma and epithelium plays an essential role in promoting invasion, progression, and metastasis [45,48,51]. However, the mechanism by which resveratrol elicits its anti-CaP progression effects remains unclear. Accordingly, we have begun to study the effects resveratrol might exert on communication between these two cell types in the tumor microenvironment. Historically, cell scatter analysis has been used to validate the activity and function of HGF using MDCK and other cell lines [21,52,53,54]. Although MDCK cells are accepted as a prototype for determining cell motility, they originate from canines and; therefore, may not be suitable for the analysis of cell motility in CaP in humans. In this study, we explored the patchy cluster growth characteristics of DU145 cells [35,36,37,38] to develop a time lapse, quantitative readout of cell motility. We showed that DU145 cells responded to CM from untreated PrSC by undergoing HGF-antibody-neutralizable cell migration (Figure 5). In addition, we also demonstrated that resveratrol inhibited cell migration and invasion using co-cultured stromal and epithelial cells in Boyden chambers—enabling evaluation of effects of resveratrol on control of tumor cell motility in a “physiologically relevant” microenvironment (Figure 2). The above findings are highly significant, showing that resveratrol blocks the acquisition of epithelial tumor cell motility by: (i) Suppressing HGF expression and secretion from cultured PrSC, and (ii) disrupting HGF signaling events in epithelial tumor cells. In summary, we have identified a chemopreventive role of resveratrol in controlling the acquisition of migratory behavior by co-regulating the growth of the stroma and epithelium and disrupting the bi-directional epithelium–stroma interplay mediated by HGF. Knowledge on how this grape-derived polyphenol influences tumor migratory phenotype is a requisite in the development of diet-based complementary strategies targeting both the microenvironment and the tumor.

## 4. Materials and Methods

### 4.1. Reagents

Resveratrol was obtained from Sigma-Aldrich (St. Louis, MO, USA). Recombinant human HGF and anti-hHGF antibody was purchased from R&D systems Inc. (Minneapolis, MN, USA).

### 4.2. Cell Culture

Human DU145 and PC-3 CaP cells were obtained from American-Type Culture Collection (ATCC, Rockville, MD, USA). Cells were maintained in RPMI-1640 media (Mediatech Inc. Pittsburgh, PA, USA) containing L-glutamine, supplemented with 10% FBS (Atlanta Biologicals Inc. Lawrenceville, GA, USA), penicillin (100 U/mL), and streptomycin (100 µg/mL) (Mediatech Inc. Pittsburgh, PA, USA). Culture media were changed every 3–4 days and cells were split once a week, as described [24]. Prostate stromal cells (PrSC) were purchased from BioWhittaker (Walkersville, MD, USA) and maintained in FGM2 Bulletkit containing the requisite cell type-specific growth factors, cytokines and supplements [55].

### 4.3. Cell Invasion Assay

Tumor cell invasion through a reconstituted basement membrane (Matrigel) was performed using the Boyden chamber (HTS multiwell^TM^ insert system with 8.0 µm pore size/PET membrane, Becton, Dickinson and Company, Franklin Lakes, NJ, USA). CaP cells were seeded onto the upper compartment (5 × 10^4^/mL, 0.25 mL/compartment), and PrSC cells (1 × 10^5^/mL, 0.5 mL/chamber) were added to the lower chamber, with or without the addition of 50 µM resveratrol. Following 10-day culture in a CO_2_ incubator, the upper and lower chambers were removed and washed with PBS, and separately fixed and stained with 1% crystal violet. Stained cells in the upper chamber represented those that did not migrate and penetrate the partitioning membrane between the upper and lower chambers. To quantitate their presence and abundance, the upper chamber was extracted with 10% acetic acid and OD570 was determined. The readout was used to calculate inhibition of cell growth and suppression of cell migration and penetration by resveratrol using the formula: 1-[(OD570 for resveratrol treated cells/OD570 for untreated cells) × 100]. The same determination and calculation was used for cells in the lower chamber, which represented both the migrated CaP and resident PrSC cells. Inhibition in response to resveratrol was calculated as follow: 1-[OD570 for resveratrol treated total cell counts _CaP+PrSC_/OD570 for untreated total cell counts _CaP+PrSC_) × 100]. Experiments were performed in duplicate.

### 4.4. Preparation of Conditioned Medium (CM) from PrSC and Assay of Secreted HGF in CM

PrSC cells were treated with different concentrations of resveratrol for 1 to 3 days. The CM was collected at different time points, centrifuged, and stored at 4 °C. The CM was used for the cell migration analysis and the amount of HGF present in CM was determined by enzyme-linked immunoassay ELISA (R&D systems Inc. Minneapolis, MN, USA). Experiments were performed in triplicate.

### 4.5. Cell Motility/Migration/Scatter Assay

Changes in morphology, motility, and clustering of DU145 in response to CM from PrSC and/or HGF were evaluated by time lapse image microscopy, using modified published procedures [18]. In brief, cells were seeded in 6-well plates with or without CM prepared from PrSC cells treated with resveratrol, as indicated above. The images of cells were captured by microscopy (Axioplan 2, Zeiss, Oberkochen, Germany) equipped with a charge-coupled device (CCD) camera (Axiocam, Zeiss). Time lapse images were collected and analyses were performed with the Adobe Photoshop that allowed frame-to-frame analysis of positions of each cell. DU145 cell movements were tracked by the time-dependent migration of cells relative to time 0 and the rate of cell motility/migration/scatter was determined. The details of Image analyses were as described in Supplementary Materials.

### 4.6. RT-PCR Analysis and Determination of Gene-Specific mRNA Expression

Total RNA was extracted from DU145 using the TriZol reagent (Invitrogen, Carlsbad, CA, USA). Isolated RNA (0.5 µg) was reverse transcribed (RT) with one-step RT-polymerase chain reaction (PCR) kit (Promega Corp., Madison, WI, USA). The PCR primer sequences of E-cadherin and GAPDH were as follows: E-cadherin, forward 5′-GCC AAGCAGCAGTACATTCTACACG-3′, reverse 5′-GCTGTTCTTCACGTGCTCAAAATCC-3′ [56]; GAPDH, forward 5′-CCACCCATGGCAAATTCCATGGCA-3′, reverse 5′-TCTAGACGGCAGGTCAGGTCCACC-3′. PCR reaction conditions for all genes were as follows: denaturation at 95 °C, 5 min, followed by 29 cycles of denaturation at 95 °C for 30 s, annealing at 60 °C for 30 s, and extension at 72 °C for 30 s. The expression of GAPDH was used as an internal control for normalizing mRNA expression results.

### 4.7. Protein Extraction and Western Blot Analysis

Control and treated cells were rinsed with ice-cold PBS and suspended in RIPA buffer (50 µl/10^6^ cells) containing 50 mM Tris, pH 7.4, 150 mM NaCl, 1 mM EDTA, 1% Triton^®^ X-100, 1% deoxycholate, 0.1% SDS, 1 mM dithiothreitol and 10 µL/mL protease inhibitor cocktail. The extracts were centrifuged and the clear supernatants were stored in aliquots at –70 °C. Protein concentrations were measured with Pierce protein assay reagent (Pierce Chem. Co., Rockford, IL, USA). For Western blot analysis, 10 µg proteins were boiled for 5 min in Laemmli buffer and separated on 10% SDS-polyacrylamide gel electrophoresis (SDS-PAGE). The gels were then transferred to nitrocellulose membranes (Schleicher and Schuell Biosciences, Keene, NH, USA) by a semi-dry transfer method. After blocking with TBST containing 5% low-fat milk, the membranes were probed for the level of expression of E-cadherin and ß-actin (Santa Cruz Biotechnology, Santa Cruz, CA, USA). All antibodies used in the experiments were diluted at 1:1000. Specific immunoreactivity was demonstrated by enhanced chemiluminescence (ECL) or color reaction using procedures detailed in the manufacturer’s protocol (Kirkegared and Perry Laboratories, Gaithersburg, MD, USA).

### 4.8. Statistical Analysis

Significance in the various cellular and biochemical parameters between control and treated samples was determined using the Student *t*-test. The results are presented as means ± SD. Results will be considered significant at *p* < 0.05.

## Supplementary Materials

Supplementary Materials can be found at https://www.mdpi.com/1422-0067/21/5/1760/s1.

## Abbreviations

AD	androgen-dependent
AI	androgen-independent
ATCC	American-Type Culture Collection
AU	arbitrary units
CaP	prostate cancer
CCD	charge-coupled device
CM	conditioned medium
ELISA	enzyme-linked immunoassay
EMT	epithelial-to-mesenchymal transition
HGF	hepatocyte growth factor
HRPC	hormone refractory prostate cancer
PrEC	prostate epithelium cell
PIN	prostatic intraepithelial neoplasia
PrSC	Prostate stromal cells
PSA	prostate specific antigen
RT-PCR	reverse transcribed polymerase chain reaction
SDS-PAGE	SDS-polyacrylamide gel electrophoresis
