# Supplementary material for: Resveratrol Suppresses Prostate Cancer Epithelial Cell Scatter/Invasion by Targeting Inhibition of Hepatocyte Growth Factor (HGF) Secretion by Prostate Stromal Cells and Upregulation of E-cadherin by Prostate Cancer Epithelial Cells
Source: Int J Mol Sci. 2020 Mar 4;21(5):1760. doi: 10.3390/ijms21051760 (PMC7084722; doi:10.3390/ijms21051760)
Supplement: Supplementary file 1 [file ijms-21-01760-s001.pdf]

Supplement methods

Procedure for creating an overlaid image to follow cell migration using Adobe Photoshop

First, acquire and open the 2 files to be merged. Designate a separate color for each of the 2 files, use the brush tool to color in the cells that will be followed. Use red for cell layer at time 0 ($Time_I$), and a green for the cell layer at a later time ($Time_F$). Once all cells have been color painted on both images, go to the latter of the two images ($Time_F$) and select the entire image by pressing "CTRL+A" or go to the "Select" menu and choose "Select All". Copy the image. Next switch to the first image ($Time_I$) and Paste the image on top of it. You will now see '$Time_F$' only. Next go to the "Layers" Menu at the right hand of the screen. If you do not see the "Layers" menu you must go to the "Window" menu at the top and select "Layers". In this menu you will see a slider bar to adjust the "Opacity" of the layer. Now with the '$Time_F$' Layer selected, adjust the opacity of the layer until both layers can be seen. Once you are certain the images are visually recognizable and presentable, flatten the images. To do this, go to the "Layer" menu at the top of the screen and select the "Flatten Image" option. This will flatten all the layers and create just one layer. Save the flattened image as a '*jpeg*'. You can now make additional adjustments on the "Levels" of the image (brightness, contrast, hue, saturation, etc…) by going to the "Image" menu at the top, then "Levels", then "Adjust Levels". Or you can simply press "CTRL + L", and this will take you to the levels adjustment screen. Save the images each time they are polished and refined. Select the entire image for import into Powerpoint or a program of your choice.

Obtaining and Plotting the Coordinates Using Photoshop and Excel

After the merged images have been obtained, the coordinates of each cell can be visually plotted as they appear in Photoshop, as follows. Go to the top menu “Window” and select “Info”. The “Info” window contains an array of information concerning color levels as well as the X and Y coordinates on a given image. Select and record the initial and final coordinates for each of the cells. Enter all coordinates in Excel and plot them on a ‘scattered line plot.’ Note that the Y coordinates are reversed in Photoshop, so the Y axis in Excel must be flipped. This process will generate a plot of the initial and final coordinates for the cells that you have marked for the migration study. Copy and paste the plot into Powerpoint.
